# Comparison of Sensorimotor Rhythm (SMR) and Beta Training on Selective Attention and Symptoms in Children with Attention Deficit/Hyperactivity Disorder (ADHD): A Trend Report

**Published:** 2015-06

**Authors:** Mohammad Reza Mohammadi, Nastaran Malmir, Ali Khaleghi, Majd Aminiorani

**Affiliations:** 1 Psychiatry and Psychology Research Center, Roozbeh hospital, Tehran University of Medical Sciences, Tehran, Iran; 2 Clinical Psychology Department, Islamic Azad University, Science and Research Branch, Tehran, Iran; 3 Biomedical Engineering Department, Islamic Azad University, Science and Research Branch, Tehran, Iran

**Keywords:** Selective Attention, ADHD Symptoms, Sensorimotor Rhythm (SMR), Beta, Neurofeedback Training

## Abstract

**Objective:** The aim of this study was to assess and compare the effect of two neurofeedback protocols (SMR/theta and beta/theta) on ADHD symptoms, selective attention and EEG (electroencephalogram) parameters in children with ADHD.

**Method:** The sample consisted of 16 children (9-15 year old: 13 boys; 3 girls) with ADHD-combined type (ADHD-C). All of children used methylphenidate (MPH) during the study. The neurofeedback training consisted of two phases of 15 sessions, each lasting 45 minutes. In the first phase, participants were trained to enhance sensorimotor rhythm (12-15 Hz) and reduce theta activity (4-8 Hz) at C4 and in the second phase; they had to increase beta (15-18 Hz) and reduce theta activity at C3. Assessments consisted of d2 attention endurance test, ADHD rating scale (parent form) at three time periods: before, middle and the end of the training. EEG signals were recorded just before and after the training.

**Result:** Based on parents’ reports, inattention after beta/theta training, and hyperactivity/impulsivity were improved after the end of the training. All subscales of d2 test were improved except for the difference between maximum and minimum responses. However, EEG analysis showed no significant differences.

**Conclusion:** Neurofeedback in conjunction with Methylphenidate may cause further improvement in ADHD symptoms reported by parents and selective attention without long-term impact on EEG patterns. However, determining the exact relationship between EEG parameters, neurofeedback protocols and ADHD symptoms remain unclear.

ADHD is considered as a widespread, durable, and neurodevelopmental disorder (1, 2). It affects approximately 5% of school-age children and 2.5% of adults (45). Despite the difference of opinions for accurate diagnosis of ADHD, inattention, hyperactivity and impulsivity symptoms are considered as key impairments. Attention refers to a complex and polyhedral process with multifaceted nature which may be the prerequisite for majority of other cognitive functions (3, 4, 5). The specificity of attention deficits to ADHD and ADHD subtypes has been mainly discussed (6, 7, 8 and 9). When applying a specific treatment for ADHD, ADHD symptoms should be considered as a spectrum which varies from hyperactivity/impulsivity to any attentional difficulties. “Selective attention” is defined as one of the main deficits in ADHD (10, 11, 12, 13), selecting the target item while attenuating irrelevant stimulus at the presence of a conflicting distracting information (14, 15, 16).

Also, the analysis of electroencephalogram (EEG) signals, as an informative quantitative method, has revealed that EEG abnormalities in children with ADHD (17, 18, 19 and 20) may reflect impairments in their cognitive functions. (21, 19 and 20) Neurofeedback (or EEG biofeedback) comes from this view that impairments in ADHD are most likely associated with problems of brain oscillations: and participants can gain voluntary control over brain activities to normalize them by taking real-time visual or auditory feedback. In the recent decades, many attempts have been made to estimate the efficacy of neurofeedback for symptom reduction and cognition enhancement in children with ADHD (23, 22, 24, 25, 26, 27, 28, 29, 44, 32 and 33). Some reports provided evidence for the long-term efficiency of neurofeedback in children with ADHD (31). However, some reports were not necessary (34). In addition, methylphenidate (MPH) as an efficacious and transient (35) treatment has become the most prescribed psychostimulant medication. However, the impact of stimulant medications on cognitive functions like selective attention is questionable (36, 37). Given the psychostimulant impact chemical of the brain and the neurofeedback regulating the cortical activation, it can be expected that using both is more effective. 

In this context, we investigated the double impact of SMR/beta neurofeedback linked methylphenidate on the performance of children with AHDH in an overall measure for selective attention (d2-test) and ADHD symptoms. 

*Neurofeedback Training*

Neurofeedback is considered as an operant conditioning of neural oscillations, in which the brain is trained to gain control over specific EEG parameters by real-time visual or auditory feedback. The desired brain activity is reinforced and undesired brain activity is inhibited. Several studies supported that neurofeedback training is a promising treatment for different disorders, especially for ADHD (46). In this study, neurofeedback training was conducted over 30 sessions; two training sessions per week, each lasting 45 minutes using biograph infinity software 5-1-4 made by the Thought Technology Company. Previous studies recommended Beta/SMR protocol as a training program by which participants could increase SMR and beta and down regulate theta. Increase in the power of SMR on C4 (based on international 10-20 system) is associated with the reduction of hyperactivity/impulsivity symptoms and facilitating thalamic inhibitory mechanisms. Also, enhancement of beta waves and decrease in excessive theta in the left hemispheric on C3 are recommended to improve attention. Hence, the training included two sections (15 sessions in each section).

The aim of the first section was to train the participants to improve the amplitude of SMR (12-15Hz) and reduce the amplitude of theta waves (4-7 Hz) on C4. In the second section, the participants were trained to increase their beta (15-18) and diminish their theta activity on C3. 

The training program was conducted opened eyes with reference placed on the near earlobe using automatic adjustment reward thresholds: 80% and 20% for reward and inhibit bands, respectively. Participants had to maintain the desired activity for two milliseconds then were reinforced by auditory or visual feedback. When they achieved the determined goal, the threshold became more difficult.

## Material and Methods

In this study, 16 children with ADHD combined type (ADHD- C), 3 girls and 13 boys, comprised the sample size. These participants received MPH and neurofeedback training (NFT). 

All of them fulfilled DSM-IV criteria for ADHD diagnosis by a child and adolescent psychiatrist which was confirmed using the ADHD rating scale. The parents of participants completed the consent form.

Inclusion criteria included: 1) age 9-15 years (to eliminate the influence of development on selective attention); 2) IQ > 90 (based on Rayven test); 3) a diagnosis of ADHD combined type (based on DSM IV criteria). Exclusion criteria were the lake of the following conditions: 1) diagnosis axis I disorders; 2) neuropsychiatric disorder; 3) neurologic disorder; 4) convulsion background. 

None of the participants had experienced cognitive training before: all of them were taking MPH. Assessment included behavioral and cognition at three times: before the start of the training, between the two phases of training, and after the end of the study. EEG analysis was performed before and after the study. Behavioral assessment was performed by ADHD rating scale, and D2 test was used to assess selective attention. 

*D2 Test*

 Concentration Endurance Test (d2 Test) was developed in Germany in 1962 and was introduced as a reliable and valid measure to estimate selective attention (38). Bagheri (2011) reported acceptable internal consistency, validity and reliability, especially for GZ and KL subscales in the Iranian population (48). It is a timed dependent test that requires the participant to discriminate the target stimuli, while similar items are presents. Items arrange in 14 lines, containing 47 characters. Subjects should check each character and tag the targets (include the letter “d” with 2 dashes both on top, bottom or one on top and one on bottom) in 20 seconds per line. Visual scanning accuracy and speed are two important outcomes. In the present study, F (the number of both omission and commission errors), GZ (the number of the processed responses including correct or false), KL (the number of correct responses minus commission error), SB (the difference between maximum and minimum responses) were measured. 

*ADHD Rating Scale*

 This scale includes 18 items to measure inattention, hyperactivity/impulsivity symptoms. The score range is from 0 to 54, a high score indicating more intensive ADHD symptoms. Faries et al., (2001) showed acceptable level of test- retest and inter-rater reliability, convergent validity and internal consistency for this scale for assessing ADHD symptoms. Moreover, the scores of this scale are comparable to scores of other validated scales like Conners questionnaire (47).

This scale was completed by parents of each participant before the beginning of the training, between the two protocols and after the end of the training. 

**Fig. 1:**
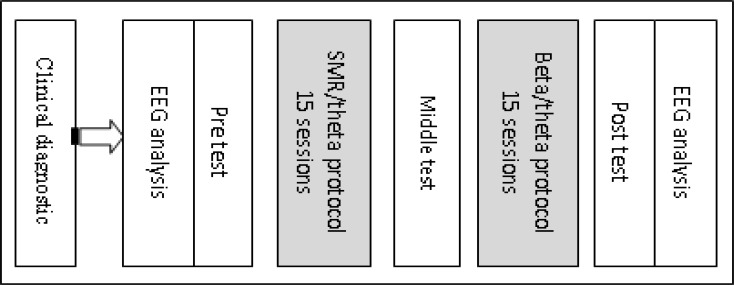
Neurofeedback was divided into two phases. Children with ADHD-C conducted first SMR/theta then beta/theta training. Behavioral and cognitive assessments were done before, between the two of the protocol and after the training. EEG analysis was performed before and after the end of the study.

In addition to measuring the IQ of the participants, we used Raven progressive matrices test for adults. Participants who were above the medium entered the study. Fig 1 shows the study design.

*EEG Recording*

**Figure 2:**
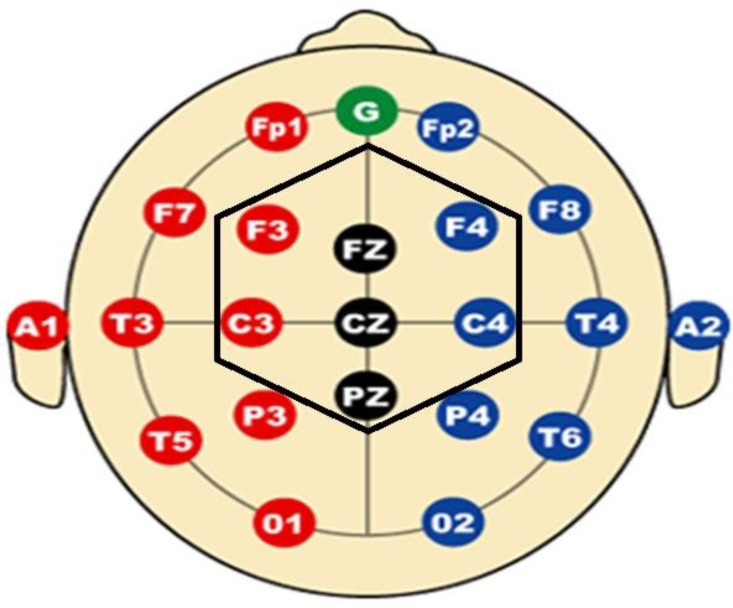
10-20 international System for EEG Recording the electrodes inside hexagonal (i.e. F3, F4, Fz, C3, C4, Cz and Pz) were used to record the signal.

 Electroencephalogram signal indicates the brain electrical activity and gives us useful information about functional status of the brain and its structural pattern. EEG was recorded using Digital EEG SD-C24 from 7 channels based on 10-20 international system (Fig. 2). Limits of band-pass filter were set to 0.1-64 Hz. The sampling rate was equal to 256 Hz for digitizing the signals. A1 and A2 channels were used for references. Recording was performed in a noiseless room at a seated position in a resting state with open eyes, and a 130- second signal was recorded for each participant before and after neurofeedback training sessions. A 40- second segment of the signal was formed from 2s epochs with minimal artifacts including EOG (electrooculogram) and EMG (electromyogram) interferences by an experienced neurologist for each participant, and spectral analysis was performed off-line.

*Data Analysis*

Data were analyzed using the Friedman test and repeated measures ANOVA, followed by Bonferroni test for pair wise comparison of related means scores between the three times of the assessment. 

Given the lack of error covariance matrix and normality assumptions for d2 scores, Freidman test was used to detect the differences for the test. The statistical analysis was conducted by SPSS (V.21), and p<0.05 value was considered significant.

The statistical analysis of EEG power spectral was performed using independent t-test (parametric test) or independent Mann-Whitney

 (non- parametric test) according to data distribution. Kolmograph-Smirnov test was utilized to evaluate the normality of the data, and the features (power spectral) with normal distribution were examined via independent t-test. Also, the features with non-normal distribution were examined via independent Mann-Whitney test. The features were considered significantly different at the level of P < 0.05. 

*Spectral Analysis of EEG Signal*

Power spectral density (PSD) of EEG signals was computed using the Welch periodogram technique which is based on Fast Fourier transform (FFT). Since the time length of our epochs was 2 seconds, frequency resolution was 0.5 Hz. Five frequency bands were extracted and their power spectral was computed: delta (0.5-4 Hz), theta (4-8 Hz), alpha (8-12 Hz), SMR (12-15 Hz), beta1 (15-18 Hz), beta2 (18-34 Hz) and gamma (34-44 Hz).

## Results

*Overall Analysis*

Demographic features are briefly illustrated in Table 1. To eliminate the effect of development on selective attention, children older than 9 years were selected. All of the participants met the ADHD-C criteria. 

**Fig3:**
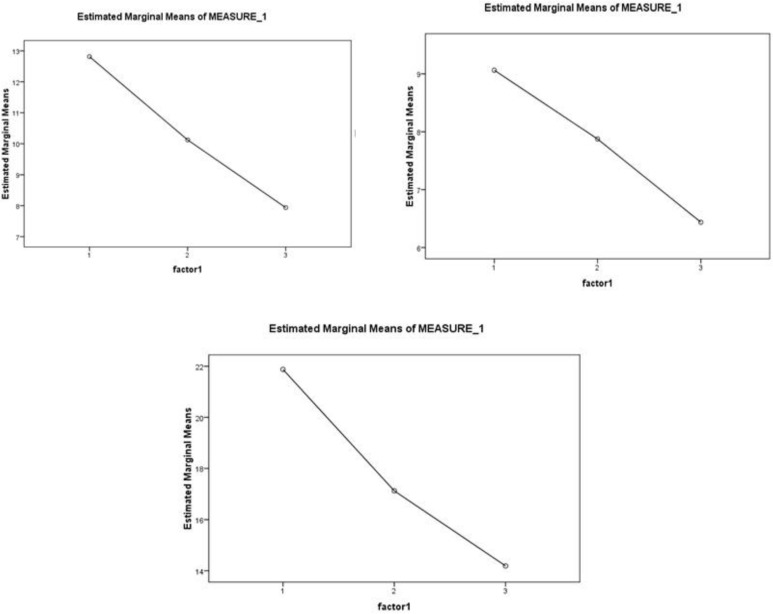
Left top row shows inattention assessment before, middle and after training. Right top row shows hyperactivity/impulsivity assessment before, middle and after training. Bottom row indicates the total score in the inattention and hyperactivity/impulsivity assessment pre-, mid- and post-training.

Means and standard deviations of all pre, middle and post measures in ADHD rating scale were calculated and presented in Table 2, and differences between assessments are demonstrated in Table 3. In addition, Figure 3 shows the trend reports of modifications in parent’s reports. 

*Behavioral Assessment*

ADHD rating scale was used to assess the effect of different protocols of neurofeedback on symptoms of ADHD, and results were analyzed using repeated measures ANOVA and Bonferroni tests (p<0.01). The results of descriptive analysis of ADHD rating scale are presented in Table 2.

Given the with-in subject effects and F score (F inattention= 12.2, F hyper/imp= 3.68, F total= 11.78,) in Table 2, the sample group showed significant differences in three levels of assessment on ADHD symptoms based on parents’ reports (p<.001).

The paired comparisons between the measures by Bonferroni test are presented in Table 3. The results of Bonferroni test showed that the difference in the total variable means in pretest and middle test was not significant (p<.001). However, the total variable mean of the post test is higher compared with the middle test. Fig 3 shows the comparison between the three times of the assessment. 

*D2 Test*

Given the lack of error covariance matrix and normality assumptions for d2 scores, Friedman test was used to detect the differences for the test. Means and standard deviations of the four subscales of d2 test in the three levels of the study were calculated by Friedman test (Table 4). Significant improvement was observed in the 3 subscales (GZ, F, KL): GZ ( χ2=14.00, p<.01), F (χ2= 12.87, p<.01), KL (χ2= 14.00, p<.01). However, no difference was observed for SB score

*EEG Analysis Results*

**Fig. 4:**
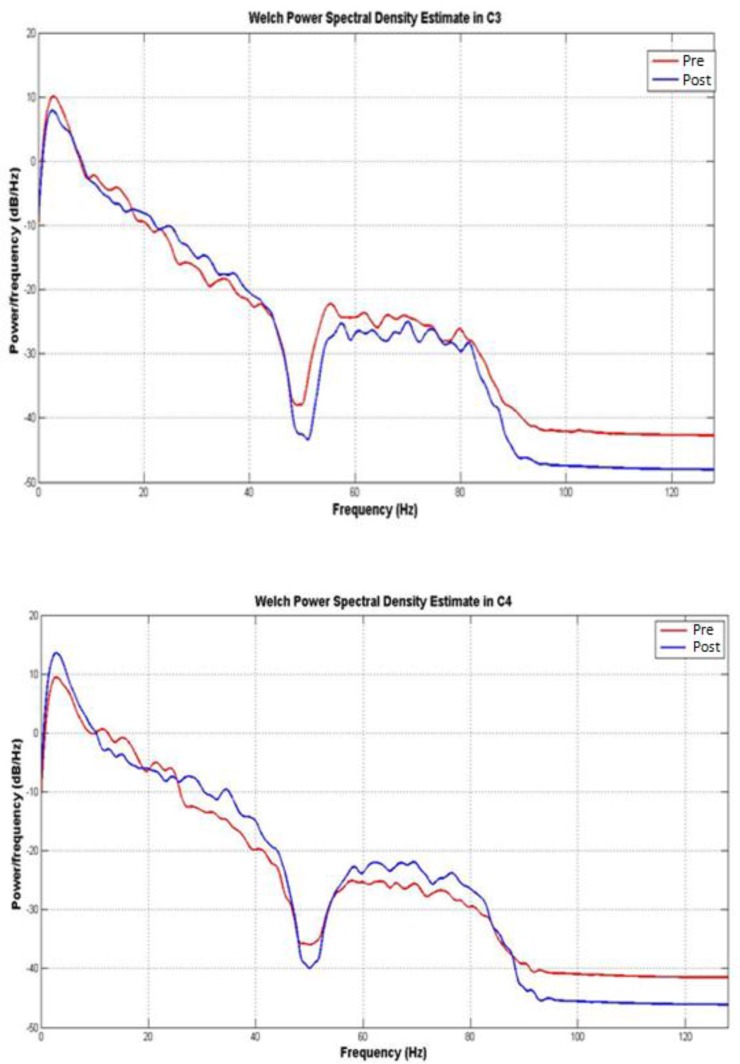
Power Spectral Density of C3 and C4 Channels before and after Neurofeedback

Figure 4 shows the PSD of EEG signal plots for C3 and C4 channels. Based on the statistical analysis, there were no significant differences between, before and after neurofeedback in all examined frequency bands in every 7 electrodes (P > 0.05). Although the differences were observed in some channels, power spectral variations in delta, theta, alpha, beta and gamma bands were not enough to be evaluated as statistically significant pre and post neurofeedback. 

**Table 1:** Demographic and Clinical Characteristics of the Experimental Group (n= 16)

	**Children with ADHD-C** **(n = 16)**
Age	10 ± 2.18
Sex (boys/girls)	11 / 3
IQ (Raven test)	113.12 ± 7.21
ADHD-RS-Parents	
Inattention	12.81
Hyperactivity/Impulsivity	9.06

**Table 2:** Summary of Repeated Measures ANOVA Results for the Inattention and Hyperactivity/Impulsivity measured by ADHD Rating Scale in the Pre-, Mid- and Post- Neurofeedback

**Behavior Ratings**	**Pre** **M (SD)**	**Middle** **M (SD)**	**Post** **M (SD)**	**SS**	**df**	**MS**	**F**	**η2**
ADHD-RS-Parent								
Inattention	12.81 (5.02)	10.12 (4.11)	7.93 (4.69)	190.79	2	97.77	12.22**	0.44
Hyperactivity/Impulsivity	9.06 (5.51)	7.87 (5.11)	6.43 (4.09)	55.29	2	27.64	3.68*	0.19
Total Score	21.87 (9.97)	17.12 (7.97)	14.18 (8.28)	481.54	2	11.78	11.78*	0.44

** 0.01,

* 0.05

**Table 3:** Summary of Changes in the Inattention and Hyperactivity/Impulsivity measured by ADHD Rating Scale in the Pre-, Mid- and Post- Neurofeedback using Prosecution Case Bonferony Test in the Neurofeedback Group (n=16)

	**Groups**	**Pre**	**Mid**	**Post**
Inattention	Pre	-	2.68	4.87**
	Mid	-	-	-2.18*
	Post			-
Hyper/Imp	Pre	-	1.18	2.62*
	Mid	-	-	1.43
	Post			-
Total Score	Pre	-	4.75	7.69**
	Mid	-	-	2.93
	Post			-

** 0.01,

* 0.05

**Table 4:** D2 Subscale Analysis by the Non- parametric Freidman Test in the Pre-, Mid- and Post- Neurofeedback for the Neurofeedback Group (n=16)

	**Pre** **M (SD)**	**Middle** **M (SD)**	**Post** **M (SD)**	**Chi-Square**	**df**	**Sig.**
GZ	287.12 (107.74)	355.75 (93.67)	333.37 (104.25)	14.00	2	0.001
F	39.00 (47.82)	14.68 (14.89)	14.00 (13.66)	12.87	2	0.002
KL	117.62 (51.25)	150.87 (43.09)	136.62 (54.13)	14.00	2	0.001
SB	15.25 (10.57)	13.00 (4.01)	11.37 (4.3)	1.20	2	0.54

## Discussion

The aim of this study was to explore the efficacy and compare both SMR/theta and beta/theta protocols to enhance selective attention and reduce ADHD symptoms; this was done in terms of EEG changes and modification in d2 attention endurance test and symptoms measures. 

Based on parents’ reports, enhanced attention was observed in ADHD children after beta training (i.e, in mid to post period of the training sessions). The results of hyperactivity/impulsivity score showed significant improvement from pre- to- post treatment, but not in pre-mid and mid-post treatment. Neurofeedback training also led to improvement in all of subscales of d2 attention endurance test except for SB score in the two phases of the training. 

Also, analysis of EEG parameters showed no significant differences in EEG power before and after training. 

It is interesting to note that MPH and neurofeedback training did not affect the power spectral of recorded signals, while d2 test and ADHD rating scale had considerable modifications toward improved performance in participants. While many attempts have been made to evaluate the efficacy of neurofeedback, little information is available about the effect of neurofeedback on EEG parameters. Lubar, 2003 (40) separated two conditions toward a response to neurofeedback: responders and non-responders, and found that EEG changes contribute to more improvement in behavioral assessment, whereas in this study this was not observed. This finding is consistent with previous studies that investigated the neurofeedback and MPH effects on EEG (21, 39). For example, Lansbergen et al., (2011) suggested that the observed behavioral improvements in neurofeedback studies may be caused by unspecific factors (e.g., expectancy, interaction therapist or just passed time) rather than by regulation in the brain oscillations. There are some explanations for our finding. First, EEG was recorded just from 7 channels, and we did not trace all changes in other channels. Second, we used automatic reward threshold adjustments in this study and it might not be as effective as manually adjusted reward threshold (34). Third, participants did not examine the specific EEG deviations before the beginning of this study. Lansbergen et al., (2011) speculated that neurofeedback may not be effective for normalized deviant EEG features. Moreover, the participants of our study were taking MPH during the neurofeedback training, and to confirm the results of EEG pattern in this study, a larger sample size may be required. 

Significant improvement of inattention in the second phase of the training provides a support for the efficacy of beta/theta training for attention enhancement. These results are consistent with previous studies which reported that beta training is associated with attention enhancement (41, 42, 43). Also, larger effect size was obtained for the inattention score than the hyperactivity/impulsivity score. Improvement in the hyperactivity/impulsivity score was observed in the end of the training, while we expected it to happen after SMR/theta protocol (first phase of training). In line with these findings, other studies have reported medium effect size for hyperactivity/impulsivity after neurofeedback training. Furthermore, participants demonstrated better performance in d2 test after the end of the training. They were able to perform faster in detecting the target stimuli with fewer mistakes after training. Indeed, they performed the task with more accuracy. Since ADHD children tend to act faster without proper accuracy, SB score (the difference between maximum and minimum responses) is offered as an indicator to show impulsivity. In the current study, there was no statistically significant improvement in this score that might be caused by the impact of taking MPH. 

These results can clarify the clinical decisions in ADHD protocol selection and improve sensitivity and specificity of the decisions about the number of sessions in a treatment setting. However, ADHD is a disorder with a spectrum of symptoms from variety of attention problems to hyperactivity/impulsivity, and these results should be interpreted with caution. 

Absence of an experimental group should be mentioned as a limitation of this study.

## Limitations

Small sample size, absence of an active control group and the follow up assessment should be mentioned as the limitations of this study. 

## Conclusion

In this study, our aim was to determine the efficacy of SMR/theta and beta/theta training and compare the results between the two phases of the training. Our study revealed that improvement in behavioral measures, especially attention, can be detected in a shorter period of training; and neurofeedback when combined with MPH can improve selective attention or ADHD symptoms (especially inattention) without long-term impact on EEG signals.

However, further studies should to be conducted to examine the long-term effects of neurofeedback training and clarify the relation between EEG patterns and behavioral or cognitive performance in neurofeedback setting.
